# PET/CT imaging in prostate cancer

**DOI:** 10.1186/1470-7330-15-S1-O15

**Published:** 2015-10-02

**Authors:** Tara Barwick

**Affiliations:** 1 Imperial College Healthcare NHS Trust and Imperial College, Division of Surgery and Cancer, UK

## 

Prostate cancer is the second most common male malignancy worldwide with a wide spectrum of biological behaviour ranging from fairly indolent disease to highly aggressive metastatic castrate resistant tumours. In the past decade there have been advances in therapies for treating advanced and recurrent prostate cancer and improved diagnostic/prognostic tools are required to refine the therapeutic approach at various stages in the management of prostate cancer patients.

For tumour staging/lesion localisation multiparametric prostate MRI is the modality of choice. Several PET tracers are proving more sensitive than conventional imaging techniques for early detection of lymph node and bone metastases. However a general limitation of PET is the spatial resolution which limits the reliability for detection of small lesions.

The use of F-18 fluoro-deoxyglucose (FDG) PET/CT in prostate cancer has been disappointing largely due to the fact that many prostate cancers have only low level glucose metabolism.

C-11/F-18 choline, markers of cell membrane metabolism, are now widely used in Europe and in certain UK centres particularly in the scenario of biochemical relapse and high risk staging with equivocal findings on conventional work-up [1-3]. However, in cases of biochemical relapse with low PSA rise and low PSA velocity, choline PET has low detection rates [4].

Prostate specific membrane antigen (PSMA), a cell surface membrane glycoprotein, is a promising target for the diagnosis and treatment of prostate cancer. Recently Ga-68 radiolabelled ligand targeted to PSMA has been introduced to a few European centres. Early data suggests this may be more sensitive than choline PET/CT in patients with biochemical failure and low PSA [5-7]. In addition it offers the opportunity for Lu-177 or Y-90 targeted radionuclide therapy.

F-18 sodium fluoride (NaF) is highly sensitive compared to bone scintigraphy with Tc99m labelled phosphates but only assess skeletal disease [8].

Other PET tracers being explored include C-11 acetate targeting lipogenesis, tracers radiolabelling bombesin and targeting gastrin-releasing peptide receptor (GRPR), amino acid transport with anti-1-amino-3-F18-fluorocyclobutane-1-carboxylic acid (FACBC ) and F-18-fluoro-5α-dihydrotestosterone (FDHT) targeting the androgen receptor [9,10].

This talk reviews the diagnostic utility of PET in prostate cancer.
